# Influence of Crowdsourcing Innovation Community Reference on Creative Territory Behavior

**DOI:** 10.3389/fpsyg.2022.902058

**Published:** 2022-05-09

**Authors:** Wei Xiao, Xiao-Ling Wang, Yan-Ning Cao

**Affiliations:** ^1^Department of Human Resource Management at Shanghai Normal University, Shanghai, China; ^2^College of Philosophy, Law and Political Science at Shanghai Normal University, Shanghai, China

**Keywords:** crowdsourcing innovation community reference, reference group, online impression management, creative territory behavior, conformity effect

## Abstract

Crowdsourcing innovation community has become an important platform for enterprises to gather group wisdom. However, how the crowdsourcing innovation community plays a reference role in creative crowdsourcing participation is unclear. Based on the reference group theory, taking online impression management as the explanatory framework, this study explores the impact of crowdsourcing innovation community reference on the creative territory behavior, and the differences in the crowdsourcing innovation community reference effect among members of different community age groups. A total 524 valid two-stage questionnaires were collected. The empirical analysis results show that under the influence of informational reference and utilitarian reference of the crowdsourcing innovation community, community members are significantly more likely to adopt acquired impression management (AIM) than defensive impression management (DIM); under the influence of value expressive reference of the crowdsourcing innovation community, the possibility of adopting DIM behavior is significantly greater than that of adopting AIM behavior; compared with DIM behavior, AIM behavior has a more inhibitory effect on creative territory behavior. Interestingly, there are different community reference effects among members of different community age groups. In particular, the positive contribution of the elder members is not as good as that of the newcomers. The above research conclusions not only confirm the influence of crowdsourcing community reference on crowd participation decision making but also provide theoretical and practical enlightenment for exploring the cooperation mechanism of crowdsourcing innovation.

## Introduction

Howe (2006) put forward the concept of crowdsourcing, which is a kind of free and voluntary outsourcing of tasks previously performed by employees to non-specific (and often large) public volunteers. With the rise of group wisdom, Tencent Cloud, Xiao MI Community, iHaier, and Dell Creative Storm Community have brought a large number of users into the crowdsourcing innovation system in an efficient and low-cost way and achieved remarkable innovation results (Afuah and Tucci, 2012; Bayus, 2013). But it is not easy for companies to operate crowdsourcing innovation communities to collect rich and valuable creatives such as product experience, improvement suggestions, and new product ideas. Hung et al. (2015) pointed out that only 3–10% of the members of large crowdsourcing communities in China will provide creativity and ideas to enterprises in crowdsourcing communities. The vast majority of crowdsourcing communities have worrying bottlenecks of creativity shortage, knowledge hiding, diving, and hitchhiking in innovation interactions (Mair and Georg, 2017). Therefore, to successfully attract and motivate the collective wisdom contribution of users, it is necessary to have a deep understanding of the role of crowdsourcing innovation communities in creative crowdsourcing participation.

In the literature, it has been found that the innovation mode of crowdsourcing does not necessarily bring about the sharing of creative resources (Michelucci and Dickinson, 2016). Creative territory behavior leads to creative resource hoarding and knowledge sharing failure (Thorgren et al., 2012). In view of individuals, territory or sharing is a social dilemma people usually face (Stouten et al., 2006). According to the theory of social resources (Peng, 2013), individuals tend to have territorial behavior tendencies and expressions toward the objects they perceive ownership. It is noteworthy that compared with other tangible resources, the ownership boundary of a creative idea is more ambiguous, flexible, and permeable, which makes the owners more likely to take creative territory behavior to establish, identify, maintain, or reconstruct their creative ideas (Avey et al., 2009; Chang et al., 2015). The formation mechanism of creative territory behavior has been explored from the perspectives of technical support (Chen et al., 2014; Xiao et al., 2022), personal motivation (Liao et al., 2013), social identity (Peng, 2013), and social network (Elderham and Da Silva, 2015). However, the above research studies mainly focused on the network node attributes and network relations at the micro-level of crowdsourcing community, and lack attention to the overall group attributes of crowdsourcing innovation communities, so the explanation of the crowdsourcing innovation cooperation mechanism is insufficient.

According to the reference group theory, the reference group is an important channel for people living in various groups as social animals to obtain interpersonal support, make an interpersonal comparison, and form subjective cognition of life (Thorgren et al., 2012). Participating in online community interaction has become a common way of life, as members of the crowdsourcing community (Cheung et al., 2014), their decision making of crowdsourcing innovation participation is bound to be influenced by the reference of the community and other members. Nevertheless, it remains to be verified whether reference group theory has explanatory power in the crowdsourcing innovation mode. Since crowdsourcing innovation community has the characteristics of anonymity, full-time and physical absence, traditional social clues, and norms such as income, status, and class are gradually declining (Cheung et al., 2011). The social relationship between makers and crowdsourcing enterprises, makers and makers in the crowdsourcing community is an impromptu, loose and borderless competition–cooperation relationship (Martin, 2016).

Benabou and Tirole (2003) pointed out that, extrinsic reward and impression management are important factors of individual economic behaviors. Impression management, which means people’s psychological tendency to be viewed positively and avoid being viewed negatively by other members of the community, has become an important motivation for users to participate in community activities (Michikyan et al., 2015). Different from open-source community, virtual brand community, and social community, the crowdsourcing innovation community has the dual attributes of innovation participation and online social networking (Yan et al., 2019). However, most scholars study internal and external motivation from the perspective of user innovation or enterprise innovation, while a few scholars study the two attributes of the crowdsourcing innovation community from the perspective of impression management (Wu and Chen, 2021; Xiao and Huo, 2021). The trend of people’s impression management from offline to online has also attracted the attention of the literature (Shi et al., 2014). However, impression management is more widely studied in the face-to-face than an online interaction mode (Kaur, 2016). There is a reasonable prospect that the relatively new variable of online impression management will provide a possible psychological explanation mechanism for understanding the influence effect of crowdsourcing innovation community reference (Al-Shatti and Ohana, 2021).

Based on the group reference theory, taking network impression management as the interpretation framework, this study constructs and verifies the theoretical model of the influence of crowdsourcing innovation community reference on creative territory behavior. This study contributes to the literature in several ways. First, by introducing the group reference theory into the research field of creative territory, the formation mechanism of creative territory behavior under the crowdsourcing innovation community reference is explored. It breaks through the limitation of previous studies focusing only on the micro-mechanism of the crowdsourcing community and provides a relatively middle-level theoretical perspective to explore the collaborative mechanism of crowdsourcing innovation. Second, since the explanatory power of reference group theory in the crowdsourcing innovation model remains to be verified, this study constructs and verifies the theoretical model of the influence of crowdsourcing innovation community reference on creative territory behavior to extend the explanatory power of reference group theory to virtual crowdsourcing community. Third, this study uses network impression management as an explanatory framework to reveal the black box of crowdsourcing creative interaction, which provides a possible psychological explanation mechanism for understanding the influence of crowdsourcing innovation community reference. Relevant research conclusions also provide management suggestions for crowdsourcing enterprises to identify and build crowdsourcing innovation communities and formulate effective incentive strategies for crowdsourcing innovation participation.

## Literature Review

### Crowdsourcing Innovation Community Reference

Reference group theory (Hyman, 1942) provides an effective theoretical perspective for studying the social psychological phenomena between individuals and groups. As social animals, people will inevitably contact and interact with different groups in daily life. Therefore, they will be affected by the tangible and intangible effects of groups. The reference group refers to the imaginary or real groups that have an important impact on individual beliefs, attitudes, or behaviors (Klandermans, 1993). When individuals express their behavior based on the views or values of a specific group, this group is the reference group. From the perspective of the extension of reference groups, reference groups may be accessible in daily life, or they may not have practical contact in the virtual network. With more and more social activities moving from offline to online, participating in online community interaction has become a way of life (Cheung et al., 2014). The research shows that the network reference group can not only have a direct impact on users’ online information collection behavior (Kim et al., 2016), tourism network use behavior (Berger and Rand, 2008), online purchase (Escalas and Bettman, 2003), but also have an indirect impact on users’ behavior through the intermediary role of variables such as trust (Racherla et al., 2012) and emotion (White and Dahl, 2006).

Considering the crowdsourcing innovation community as the reference group, and focusing on the comparison or reference framework provided by the network reference group for its members’ attitudes, values, and behavior decisions, this study aims to explore the impact of crowdsourcing innovation community reference on community members. By recognizing the reference group as a multi-dimensional construct (Escalas and Bettman, 2003; Pechmann and Wang, 2010), crowdsourcing innovation community reference can be summarized as informational reference, utilitarian reference, and value expressive reference. Informational reference refers to the information from crowdsourcing community and other members’ help to improve the cognitive ability of community members. Utilitarian reference refers to the behavior that community members take to meet the expectations of the group to obtain the appreciation of the crowdsourcing community or avoid punishment. Value expressive reference comes from the internalization of group values and norms by community users.

### Online Impression Management

Impression management is the internal consciousness and motivation of individual self-image display and catering to others’ recognition of self-image (Viswesvaran et al., 2001; Bao et al., 2010). With the continuous development of Internet technology, many social activities begin to be transferred to the network, which makes the formation and effectiveness of interpersonal impressions divorced from the real social scene. Michikyan et al. (2014) put forward the concept of online impression management and pointed out that to strengthen or build a self-image completely different from the real society, Internet users will adopt different impression management strategies on various real name or non-real name network platforms. Unlike WeChat moments, which focuses on social networking with acquaintances, Internet platforms and media such as microblog, WeChat group, and crowdsourcing communities enable netizens to choose, contact, and communicate with people who have common hobbies, attitudes, and values (Wu and Zheng, 2019). In the process of multiple community exchanges and interactions such as likes, comments, and posts through crowdsourcing communities, makers do form self-concept through role-playing and identity construction, especially making ideal-self and virtual-self visible (Kozinets et al., 2008).

According to the differences in causes and functions, impression management is divided into acquired impression management (AIM) and defensive impression management (DIM) (Jain, 2012). AIM aims to make others view their efforts positively and seek recognition by presenting positive aspects of themselves. DIM aims to avoid others’ negative views of themselves by weakening their shortcomings. In the crowdsourcing innovation community, people want to get a positive evaluation and do not want to get a negative evaluation. Therefore, they will control or manage their online impression through knowledge and creative activities (Ramaswamy, 2005). However, due to different motives, some users may adopt AIM, while others adopt DIM. When users want to repair or improve their reputation, they will adopt AIM strategies, such as actively undertaking community tasks, participating in posting, and showing their innovation ability. In case of negative events, DIM strategies shall be adopted to weaken their shortcomings or avoid others’ negative views of themselves, such as denial, defense, apology, compensation, correction, and deletion of posts.

**FIGURE 1:**
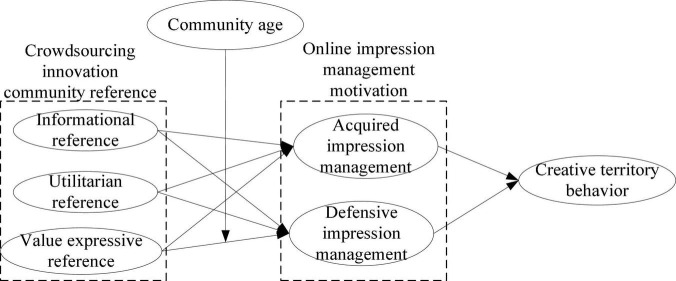
Theoretical model of the relationship between crowdsourcing innovation community reference and creative territory behavior.

Based on the group reference theory, taking network impression management as the interpretation framework, the theoretical model of the influence of crowdsourced innovation community reference on creative territory behavior was constructed as shown in Figure 1.

### Crowdsourcing Innovation Community Reference and Online Impression Management

#### Informational Reference and Online Impression Management

Informational influence is mainly manifested in that individuals can get information related to product innovation and creative experience by participating in the group interaction and communication of crowdsourcing community (Thorgren et al., 2012). Usually, individuals expect to have enough information before making decisions. Since the environment is full of various uncertainties, makers often collect a large amount of relevant information, ideas, and knowledge by paying attention to the words, pictures, and videos shared by online communities, to acquire innovation knowledge and improve product creativity skills.

In daily community life, users often contact and communicate with various reference groups. In all kinds of intentional or unintentional interactions, users get a lot of information. In the process of crowdsourcing, heterogeneous knowledge and creativity wander and couple to generate new information (Cheung et al., 2011). As useful information, the behaviors, ideas, and opinions of the community and its members are beneficial to improve the cognitive ability of individual makers and drive individual makers to adopt AIM behavior. From the perspective of social capital, the mutually beneficial relationship between knowledge co-creators can promote the formation of an emotion-based trust relationship, so as to increase the quantity and quality of mutual knowledge sharing (Brown et al., 2014). Thorgren et al. (2012) also believed that the interdependent cooperation parties are more inclined not to protect their knowledge, but to share all kinds of knowledge with a strong will and a more open mind, and ensure that each other can absorb it.

Therefore, this study puts forward the following hypothesis:

**H_1a_:** Information reference is positively associated with acquired impression management.

**H_1b_:** Information reference is negatively associated with defensive impression management.

#### Utilitarian Reference and Online Impression Management

The normative influence of the reference group refers to the tendency to meet the expectations of others (Burnkrant and Cousineau, 1975). Among them, utilitarian reference focuses on individuals’ compliance with the expectations of others to gain rewards or avoid punishment (Bearden et al., 1989). For the crowdsourcing innovation community members, participating in crowdsourcing innovation has two utilitarian motives: ability display and self-learning. First, for reasons such as dissatisfaction with existing products or wanting to improve products according to their wishes, users will interact with enterprises through a convenient crowdsourcing innovation community and participate in enterprise value co-creation (Ramaswamy, 2005). Second, for users who aim at learning, participating in crowdsourcing innovation actively is also the best choice. Rioux and Penner (2001) found that motivated by positive impression management motivation, individuals who want to improve their self-image are usually more willing to take risks, put forward positive suggestions, or actively provide help when other community members need it.

When individuals pay attention to the dynamics of the community and other members, they will take it as the reference object for their participation in decision making in crowdsourcing community creative activities. Syn and Oh (2015) pointed out that social participation is a powerful utilitarian driving force to promote members’ knowledge sharing. Geng and Shen (2019) believed that utilitarian social expectation is one of the most important motivations for users to participate in knowledge sharing. When individuals choose to adapt to the default standards and norms of the group, make their attitudes and values converge with the group, and express their recognition and love for others in the community to integrate into the group, utilitarian influence will occur. Users’ public self-expression and display in the community is an important positive means of relationship promotion, and stronger self-presentation motivation will correspondingly cause more positive feedback from other users (Bareket-Bojmel et al., 2001). Therefore, under the utilitarian reference of the crowdsourcing innovation community, it is a common impression management behavior for members to express positive emotions to the greatest extent to present and maintain a positive self-image.

Therefore, this study puts forward the following hypothesis:

**H_2a_:** Utilitarian reference is positively associated with acquired impression management.

**H_2b_:** Utilitarian reference is negatively associated with defensive impression management.

#### Value Expressive Reference and Online Impression Management

Value expressive reference emphasizes the value cognition and emotional dependence of individuals on their special social group identity (Bass, 1985). Generally speaking, when facing a certain society or group, people will refer to the characteristics of a specific society or group to determine their belonging and make corresponding contributions to the community, to obtain the recognition of the community and other members. Pagliaro et al. (2010) showed that the group pressure caused by the behavior of others in the community will make members worry that if they cannot be consistent with the behavior of others, they will not be recognized by the group or even excluded. Chen and Chen (2017) studied the tourism online community and found that customers’ recognition will significantly affect their value co-creation behavior. It was believed that in China’s society dominated by “Guanxi” and “Mianzi,” the identity of an insider is valued by members (Li et al., 2017; He et al., 2019).

In the context of a virtual online community, individual identity is no longer limited by traditional social reference dimensions (such as class, status, bureaucracy), and their cooperative innovation behavior is more affected by trust (Lee et al., 2013), value perception (Martin, 2016), and sense of belonging (Nambisan and Baron, 2010). Although a crowdsourcing innovation community is an informal and loose tribe, its members still pursue a kind of community identity (Tsang et al., 2013). Grant and Mayer (2009) found that value expressive reference has a positive impact on affinity organizational citizenship behavior, but has a negative impact on challenging organizational citizenship behavior. The characteristics of the crowdsourcing community, such as anonymity, full-time, and few constraints (Cheung et al., 2011), make people in a “streaking” state, so the value expression of every word and action in the community is likely to be amplified and misinterpreted. It can be seen that under the value expressive reference of a crowdsourcing innovation community, members need to be more careful to maintain a balance between advantages and credibility in the process of impression construction. If individuals pay too much attention to the advantages of impression and earn “Mianzi” too actively (Tsang et al., 2013), they might be counterproductive, that is, they cannot be recognized by the community.

Therefore, this study puts forward the following hypothesis:

**H_3a_:** Value expressive reference is negatively associated with acquired impression management.

**H_3b_:** Value expressive reference is positively associated with defensive impression management.

### Moderating Effect of Community Age

If different indicators of community reference have different effects on impression management, do these referential effects remain stable across different groups? Empirical studies in the field of consumption have found that brand age will affect consumers’ self-interpretation, and then affect the attribution of consumers’ reference group and consumers’ brand identity (Stephens et al., 2007). Less experienced consumers prefer independent self-interpretation, while experienced consumer groups prefer interdependent self-interpretation (Kraus and Keltner, 2009). Similar to the consumer scene, in the workplace, elder employees generally have the characteristics of abiding by norms, obeying authority and system, while new employees have the characteristics of pursuing self-independence and distinctive personalities (Grant and Mayer, 2009). Bolino et al. (2015) pointed out that the behavior of impression management requires the consumption of cognitive resources, emotional resources, and physical strength of the behavior subject. Since the new and elder members have different impression management motives, their impression management behavior is likely to be different.

For elder members, first, may have gained a certain community reputation and formed a relatively fixed community impression, so it is difficult for them to improve an individual image through general innovative participation behavior (Bolino et al., 2015). Second, there may be job burnout, which is increasingly tired, sleepy, and even tired of community innovation contribution. Third, they may have exhausted their talents, and the difficulty of innovation is relatively high. For newcomers, the positive images such as community reputation and community status are blank, and they have expectations for the future. Second, new members usually bring new heterogeneous resources such as knowledge, thinking, and values (Brown et al., 2014). Finally, just as the so-called “newborn calves are not afraid of tigers,” the enthusiasm and vitality of new members is a contribution to innovation performance. It can be seen that the longer the community age of the crowdsourcing members are, the more cautious and negative they will be about participating in crowdsourcing innovation. The shorter the community age of the members, the more positive they will be to participate in crowdsourcing innovation.

Therefore, this study puts forward the following hypothesis:

**H_4a_:** Community age positively moderates the relationship between crowdsourcing innovation community reference and defensive impression management.

**H_4b_:** Community age negatively moderates the relationship between crowdsourcing innovation community reference and acquired impression management.

### Online Impression Management and Creative Territory Behavior

The performance prediction effect of impression management is context dependent. DIM causes damage to the public social environment, while AIM promotes the public social environment (Delgado-Rodríguez et al., 2018). Rioux and Penner (2001) pointed out that if the two impression management motives of acquisition and protection can be effectively distinguished, their impact on user behavior may be clearer. Usually, individuals have an exclusive propensity to possess the perceived object, that is, what people usually say “this is mine, not yours.” Online impression management, as an important part of personal self-image management in the network situation, especially the strong desire for recognition and the fear of losing reputation, plays an important role in the online interaction among community members. This study agrees with Rioux and Penner (2001) and believes that in the crowdsourcing community with many complex interpersonal interactions, the relationship between impression management strategy and creative territory behavior is multidimensional.

Members driven by AIM hope to prove to other community members that they are willing and able to implement innovation activities through the sharing or contribution of knowledge and creativity, even if this is not what they really want to do (Nambisan and Baron, 2010). Positive impression management motivation can accelerate the process of group assimilation or alienation because few people in the group are willing to develop relationships with members with poor image and disrespect (Tsang et al., 2013). Therefore, they are more inclined to win a good reputation and appreciation in the community through creative contribution and sharing. On the contrary, community members with DIM motivation lack the motivation to establish a self-image consistent with the group identity. Under the threat of negative evaluation, they rarely take the initiative to show and express themselves in the group. They usually choose to dive or free ride in crowdsourcing community innovation activities, or wrap themselves in an information “cocoon room” (Lee et al., 2013; He et al., 2020). To sum up, for DIM, defensive behaviors such as deleting posts or carefully following posts will promote the formation of creative territory. For AIM, whether it is for positive self-improvement or to meet the expectations of the crowdsourcing community, it can inhibit the creative territory behavior of community members.

Therefore, this study puts forward the following hypothesis:

**H_5a_:** Defensive impression management positively associates with creative territory behavior.

**H_5b_:** Acquired impression management negatively associates with creative territory behavior.

## Materials and Methods

### Sample and Data Collection

The data collection of this study is based on the professional data research platform named Questionnaire Star. Members from 15 crowdsourcing innovation communities such as Tencent Cloud, Alibaba Cloud, Xiao MI Community, iHaier, and Dell Creative Storm Community were selected as subjects. The community members who are active and influential in crowdsourcing innovation activities are invited to fill in the questionnaire, and the reward of 100% winning and a random amount was provided. The survey data of this study was collected in two stages. The first stage (from 1 December 2020 to 15 December 2020) mainly collects the basic information of subjects (such as gender, age, education level, position, and community age) and the data of crowdsourcing innovation community reference. In the second stage (from 1 September 2021 to 15 September 2021), participants in the first stage were invited to evaluate online impression management and creative territory behavior.

To facilitate the data matching of the two-stage questionnaire survey, each subject’s questionnaire is given a number. The 232 questionnaires that only participated in the first stage and were missing in the second stage were excluded, and a total of 563 questionnaires were recovered. Furthermore, after eliminating the invalid questionnaires with the “Z character” rule and missing more than 10%, 524 valid questionnaires were finally obtained, and the effective recovery rate of the questionnaire was 96.63%. The proportion of male and female samples was 53.5 and 46.15%, respectively. The proportion of samples of different ages was 30.77, 42.66, 24.83, and 1.75%. The proportion of samples with different educational backgrounds was 20.98, 50.7, and 21.33%. The proportion of samples of different positions was 28.32, 23.08, 34.27, and 14.34%.

### Variables and Measurement

We used the 10-item scale developed by Escalas and Bettman (2003) to measure the crowdsourcing innovation community reference, and revise the expression of the scale according to the characteristics of the crowdsourcing innovation community, items including, “if community members make serious improper remarks, they will be excluded or disqualified from community membership.” We used a 13-item scale developed by Bolino et al. (2015) to measure online impression management, items including, “when other members of the community have an adverse impression on me due to my post, it bothers me.” For the measurement of creative territory behavior, referring to the measurement scale of territory behavior of non-physical objects by Avey et al. (2009), and combing with the situational characteristics of crowdsourcing creativity, the relevant expression of the scale was modified, four items including, “I feel I need to protect my creative ideas from being used by others.”

The above variables are latent variables, Likert-7 scale (1 = totally disagree, 7 = totally agree) was used for the corresponding measurement items. As an explicit variable, the community age is set as follows: 1 = less than 6 months; 2 = 7–12 months; 3 = 1–2 years; 4 = 2–5 years; 5 = more than 5 years. In addition, referring to previous research on crowdsourcing innovation community, gender (1 = male, 2 = female), age (1 = 19–20 years, 2 = 21–30 years, 3 = 31–40 years, 4 = 41–50 years, 5 = more than 50 years), education level (1 = high school/technical secondary school and below, 2 = College, 3 = undergraduate, 4 = master and above), and position (1 = ordinary employees, 2 = grass-roots managers, 3 = middle managers, 4 = senior managers) were used as control variables in this study.

### Common Method Variance

Several procedures are used to minimize the impact of common method bias. First, a statement is provided at the beginning of the questionnaire to explain the research purpose and ensure the anonymity of the answers. Second, in the introduction, it is pointed out that there is no right or wrong answer to reduce the anxiety of the respondents. Third, the items belonging to the same construct and dimension will not appear at the same time. Fourth, the order of items in each questionnaire is as different as possible.

The following three statistical tests show that there is no serious common method bias in the questionnaire measurement. First, the exploratory factor analysis (EFA) was used to analyze all the items in the questionnaire by Harman single factor method. When a factor accounted for more than 50% of the variance of the variable, there was a common method deviation. The 30 measurement items were analyzed by unrotated principal components factor analysis. All measurement items were aggregated into 6 factors with eigenvalues greater than 1. The first factor explained 29.13% of the variation and less than 40%, indicating that there was no high explanation rate of the variance of a single factor. Second, whether only one factor can be extracted from the sample data is tested. The results show that the hypothetical theoretical model can distinguish significantly (χ^2^/df = 153.5, *p* < 0.001) from the single-factor model, and has a higher fitting ability. Third, the common method factor is added to the structural equation model as a potential variable, and the change of the structural equation model-fitting after adding the potential variable is compared. The test results show that the fitting degree of the model to the data was not significantly improved after adding the common method deviation factor (Δχ^2^/Δdf = 8.76).

## Results

### Reliability and Validity Test

SPSS 24.0 was used to test the reliability of the sample data to judge the stability of the scale (see Table 1). The results show that Cronbach’s α coefficients of all the variables are greater than 0.8. Therefore, the scale has good internal consistency. All latent variables passed KMO sample measurement and Bartlett’s spherical test, which is suitable for confirmatory factor analysis. The results of factor analysis show that the measurement items of the same variable are distributed in the same factor. The factor loads of all variables exceed the acceptable critical value of 0.5, and the minimum factor load is 0.681.

**TABLE 1:** Confirmatory factor analysis results.

Variables	Factor loading	Cronbach’s α	KMO
Informational reference	0.743	0.868	0.912
Utilitarian reference	0.760	0.902	0.916
Value expressive reference	0.681	0.875	0.908
Acquired impression management	0.851	0.932	0.872
Defensive impression management	0.773	0.839	0.849
Creative territory behavior	0.898	0.895	0.746

Amos24.0 was used for model fitting validity analysis in this study (see Table 2). It can be seen that the fitting effect of the sample data on the hypothetical model is ideal, and the verification of the research hypothesis can be carried out. In addition, this study used the average variance extracted (AVE) value and combined reliability (CR) value to test the convergent validity of the research data. The lowest CR of all variables was 0.756, exceeding the critical value of 0.7. According to the test results of the AVE, the AVE values of all variables are greater than 0.5, which shows that the quality of the model is good. At the same time, the square root of the AVE value is higher than the correlation coefficient among variables, providing adequate discriminant validity.

**TABLE 2:** Model’s fitting parameters of this study.

Model fitting index	χ ^2^/df	GFI	CFI	AGFI	RMSEA
Ideal range	≤3	≥0.9	≥0.9	>0.5	<0.08
Model fitting value	2.448	0.908	0.907	0.761	0.075

*GFI, goodness-of-fit index; CFI, comparative fit index; AGFI, adjusted goodness-of-fit index; RMSEA, root mean square error of approximation.*

### Hypothesis Test

#### Crowdsourcing Innovation Community Reference and Online Impression Management

Referring to the relationship between crowdsourcing innovation community reference and online impression management (AIM/DIM), the test was divided into two steps: (1) regression analysis of all control variables; (2) the control variables were added, and the independent variables were analyzed by AIM/DIM.

As is shown in Table 3, first, gender, age, education, and position have no significant impact on AIM and DIM. Second, informational reference has a significant positive impact on AIM (M_2_, β = 0.633, *p* < 0.001), and has a significant negative effect on DIM (M_6_, β = −0.371, *p* < 0.001), so H_1*a*_ and H_1*b*_ was verified. Third, utilitarian reference has a significant positive impact on AIM (M_3_, β = 0.580, *p* < 0.001), and has a significant negative effect on DIM (M_7_, β = −0.394, *p* < 0.001), so H_2*a*_ and H_2*b*_ was verified. Finally, value expressive reference has a significant negative impact on AIM (M_7_, β = −0.117, *p* < 0.01), and has a significant positive effect on DIM (M_8_, β = 0.148, *p* < 0.001), so H_3*a*_ and H_3*b*_ was verified.

**TABLE 3:** Regression analysis results of the relationship between crowdsourcing innovation community reference and online impression management (AIM/DIM).

Variables	AIM				DIM			
	M_1_	M_2_	M_3_	M_4_	M_5_	M_6_	M_7_	M_8_
Gender	0.040	–0.061	–0.048	–0.009	–0.146	–0.087	0.087	–0.084
Age	–0.035	–0.023	–0.079	–0.070	–0.042	–0.049	0.012	0.003
Education	–0.152	–0.105	–0.145	–0.130	–0.035	–0.063	0.040	–0.063
Position	0.032	0.013	0.030	0.055	–0.030	0.008	0.001	–0.030
Informational reference		0.633***				−0.371***		
Utilitarian reference			0.580***				−0.394***	
Value expressive reference				−0.117**				0.148***
Δ*R*^2^	–0.004	0.214	0.165	0.032	–0.004	0.205	0.216	0.170
*F*	0.7	15.0***	11.2***	2.7***	0.7	14.3***	15.2***	11.6***

***p < 0.001; **p < 0.01. AIM, acquired impression management; DIM, defensive impression management.

#### Moderating Effect of Community Age

The moderating effect of community age was tested in three steps: (1) AIM/DIM was used to conduct regression analysis on crowdsourcing innovation community reference; (2) the moderating variable (community age) was added for regression analysis; (3) the interaction items after centralized treatment (crowdsourcing innovation community reference × community age) were added for regression analysis. The results are shown in Table 4.

**TABLE 4:** Hierarchical regression analysis results of the moderating effect of crowdsourcing community members’ community age.

Variables	AIM			DIM		
	M_1_	M_2_	M_3_	M_4_	M_5_	M_6_
Crowdsourcing innovation community reference	0.696***	0.574***	0.566***	−0.393**	−0.346**	−0.327***
Community age		0.410***	−0.421***		0.190***	0.199***
Crowdsourcing innovation community reference × community age			−0.048*			0.082*
Δ*R*^2^	0.118	0.334	0.347	0.116	0.261	0.267
*F*	35.5***	65.5***	43.7***	34.9***	46.3***	32.3***

***p < 0.001; **p < 0.01; *p < 0.05. AIM, acquired impression management; DIM, defensive impression management.

As shown in Table 4, the minimum *F* value of M_4_–M_6_ is 32.3 (*p* < 0.001), indicating that there is a significant linear relationship in the model. The corresponding Δ*R*^2^ are 0.116, 0.261, and 0.267, indicating that the explanatory power of the model is improving, and the latter model is better than the former model. In M_4_, community age and interaction item (crowdsourcing innovation community reference × community age) were added in turn. As in M_5_ (β = 0.190, *p* < 0.001), and M_6_ (β = 0.082, *p* < 0.05), the results showed that the interaction term had a significant positive moderating effect on DIM, so H_4*a*_ was verified.

Similarly, the minimum *F* value of M_1_–M_3_ is 35.5 (*p* < 0.001), indicating that there is a significant linear relationship in the model. The corresponding Δ*R*^2^ are 0.118, 0.334, and 0.347, indicating that the explanatory power of the model is improving, and the latter model is better than the former model. In M_1_, community age and interaction item (crowdsourcing innovation community reference × community age) were added in turn. As in M_2_ (β = −0.410, *p* < 0.001) and M_3_ (β = −0.048, *p* < 0.05), the results show that the interaction term has a significant negative moderating effect on AIM, so H_4*b*_ is verified.

**FIGURE 2:**
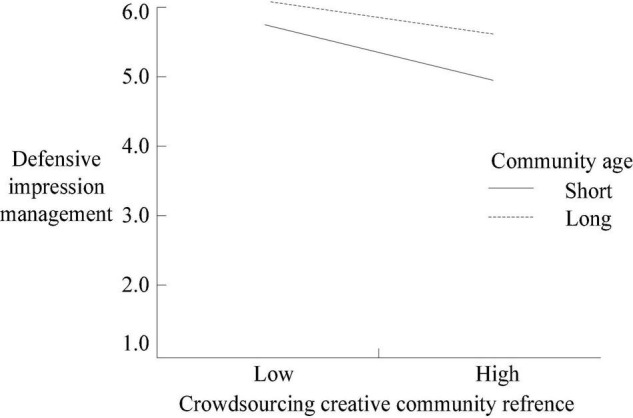
The moderating effect of community members’ community age (defensive impression management as a moderating variable).

**FIGURE 3:**
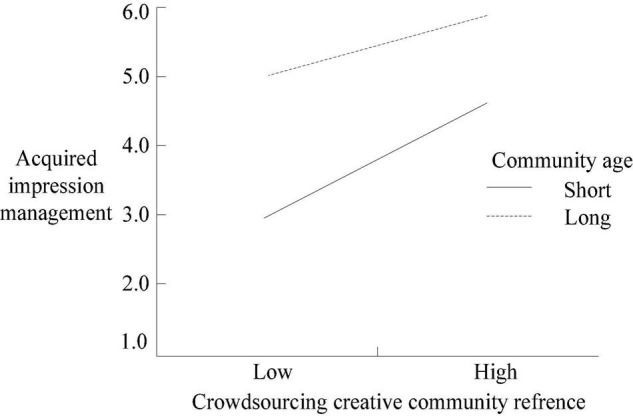
The moderating effect of community members’ community age (defensive impression management as a moderating variable).

Furthermore, by adding and subtracting 1 SD from the average community age of community members, the samples were divided into two groups: long community age and short community age. The moderating effect map was drawn as seen in Figures 2, 3). As shown in Figure 2, the community age of community members positively moderates DIM, the slopes of long and short community age curves are negative, and the slope of a long community age curve is significantly lower than that of the short community age curve. As shown in Figure 3, the community age of community members negatively moderates AIM, and the slope of the short community age curve is significantly greater than that of the long community age curve.

#### Online Impression Management and Creative Territory Behavior

To test the impact of online impression management (AIM/DIM) on crowdsourcing members’ creative territory behavior, the dependent variable creative territory behavior was regressed to acquired/DIM. It can be seen from Table 5 that AIM (*p* < 0.001, β = −0.201) has a significantly negative impact on creative territory behavior, and DIM (*p* < 0.001, β = 0.346) has a significantly positive impact on creative territory behavior, so H_5*a*_ and H_5*b*_ is verified. The reason why DIM has a significant positive impact on creative territory behavior may be that in the crowdsourcing innovation community, conservative behaviors such as apology, compensation, and correction are not beneficial to the creative contribution, while behaviors such as deleting posts, denying, and defending will have a greater negative effect.

**TABLE 5:** Regression analysis results of the relationship between AIM/DIM and creative territory behavior.

Variables	Creative territory behavior
AIM	−0.201***
DIM	0.346***
Δ*R*^2^	0.137
*F*	21.3***

***p < 0.001. AIM, acquired impression management; DIM, defensive impression management.

## Discussion and Conclusion

### Conclusion

This study introduces the reference group theory into the research field of crowdsourcing innovation. Taking online impression management as the explanatory framework, this study constructs and verifies the theoretical model of the impact of crowdsourcing innovation community reference on the creative territory behavior. The conformity effect, which is what we usually call “following the crowd,” was confirmed in crowdsourcing. When individuals are influenced (guided or exerted pressure) by the reference group, their decision-making behavior will change in the direction consistent with the majority or mainstream opinion of the community. It is believed that only by providing effective reference strategies for crowdsourcing innovation communities, activating the positive/active online impression management motivation of makers, can crowdsourcing enterprises break the psychological ownership line of maker members’ creativity, alleviate their creative territory behavior, and improve crowdsourcing innovation performance finally.

### Theoretical Implication

By introducing the reference group theory into the research field of crowdsourcing innovation, this study breaks through the limitation of previous studies focusing only on the micro-mechanism of the crowdsourcing community, and provides a relatively middle-level theoretical perspective to explore the collaborative mechanism of crowdsourcing innovation. First, the informational reference and utilitarian reference of the crowdsourcing innovation community have a significant positive impact on AIM and a significant negative impact on DIM. The value expressive reference of the crowdsourcing innovation community has a significant negative impact on AIM and a significant positive impact on DIM. This is consistent with the research conclusions of other reference situations. For example, Geng and Shen (2019) found that informational reference and utilitarian reference are significantly positively correlated with users’ willingness to share knowledge from different cultural perspectives, while insider identity perception has a positive impact on employees’ voice (Li et al., 2017). According to the group reference theory (Hyman, 1942; Kim et al., 2016), human beings, as a social animal, need to have a sense of belonging to a specific group and tend to identify and attach to the members of the group. More importantly, following the transformation of people’s social interaction principle from “social survival” to “community survival” (Heinonen and Strandvik, 2015; Puschmann and Rainer, 2016), this study attempts to extend the explanatory power of reference group theory to crowdsourcing innovation communities. The above research results provide a relatively middle-level theoretical perspective for crowdsourcing enterprises to effectively stimulate group wisdom.

Second, this study uses online impression management as an explanatory framework to reveal the black box of crowdsourcing creative interaction, which provides a possible psychological explanation mechanism for understanding the influence of crowdsourcing innovation community reference. It was found that AIM has a significant inhibitory effect on the creative territory behavior of the crowdsourcing innovation community, while DIM has a positive incentive effect on the creative territory behavior. Therefore, the crowdsoucing innovation community reference can indirectly affect the creative territory behavior by stimulating the online impression management motivation and behavior of community members. This study not only provides a research idea for the future in-depth discussion on how to reduce or even reverse the creative territory behavior of its members in the crowdsourcing community dominated by the independent innovation of individual makers (Al-Shatti and Ohana, 2021) but also inspires more research of crowdsourcing innovation to highlight the subject identity and initiative of the majority of makers to the greatest extent, to reshape the cultural capital in creative labor (Liang, 2015; He et al., 2021).

Third, this study also verified the moderating effect of community age in the relationship between crowdsourcing innovation community reference and online impression management. The longer the community age of crowdsourcing innovation community members, the stronger the motivation for DIM, and the shorter the community age, the stronger the motivation behavior for AIM. The traditional view holds that elder members have more experience and ability in innovation performance (Chen and Chen, 2017; Tang et al., 2019), but this study shows that in the crowdsourcing innovation community with the dual attributes of group innovation and online social networking, the elders show fewer positive characteristics than the newcomers, such as job burnout and worldly sophistication. This provides theoretical support for enterprises to spit out the elder and accept the new in the community.

### Practical Implication

First, crowdsourcing community information management. This study proves that maker members prefer to adopt AIM behavior under the informational reference of the crowdsourcing innovation community. Since general users are limited to the lack of professional knowledge and are difficult to participate in deep innovation. Crowdsourcing enterprises can conduct innovation-oriented online interactions with customers to guide users to participate in innovation. Furthermore, maker members who pursue value co-creation generally have the psychology of reciprocity. Enterprises can regularly share relevant information and knowledge, or organize experts to answer members’ questions in the community in time to help maker members grow.

Second, crowdsourcing community social management. This study proves that maker members prefer to adopt AIM behavior under the utilitarian reference of the crowdsourcing community. Therefore, crowdsourcing enterprises can design effective user incentive mechanisms to strengthen and maintain the good image and status of users who actively participate in the crowdsourcing community. Because it is easier for people to choose to contact and refer to people with the same characteristics as themselves, enterprises can give users who actively participate in innovation the same title or grade to enhance the reference value of these users. Combined with the strong comparison psychology of Chinese people, enterprises can stimulate users’ mentality of being unwilling to be behind others and encourage users to actively look for the possibility of creativity.

Third, crowdsourcing community standard management. This study shows that under the value expressive reference of the crowdsourcing innovation community, users tend to adopt DIM behavior, which has a certain inhibition on the creative contribution of the crowdsourcing innovation community. Enterprises can create a positive innovation atmosphere in the crowdsourcing innovation community and improve users’ cognitive threshold of group requirements, to stimulate community users to actively join and improve their creative performance. Information asymmetry makes it difficult to distinguish between good actors and good soldiers, so the standard for the community to confirm good should be higher than that for bad. Enterprises can set up community user levels according to users’ creative contributions. When other users actively participate in crowdsourcing innovation, users who prefer DIM behavior are forced to participate in innovation for fear of being labeled or excluded.

Fourth, crowdsourcing community member growth management. This study shows that in the crowdsourcing innovation community, community age positively regulates DIM behavior and negatively regulates AIM behavior. Therefore, attracting new members and eliminating some elder members has become a realistic choice for enterprises. When designing various incentive measures, the community annual leave can be set as a negative index. Take various ways to encourage more users to join the crowdsourcing innovation community.

### Limitations and Future Research Directions

This study also has some limitations, which need to be improved in future research. First, this study introduces the reference group theory into the research field of crowdsourcing innovation for the first time to explore its impact on the creative territory behavior of crowdsourcing community members. In the future, the perspective of reference group theory can be used to explain more crowd-based innovation dilemmas (such as competition vs. cooperation, class vs. equality, egoism vs. altruism), and provide more possible solutions to the tragedy of the commons in the field of crowdsourcing. Second, this study focuses on the different influence dimensions of crowdsourcing innovation community reference. Other characteristics of crowdsourcing innovation communities, such as crowdsourcing innovation community support, can be considered in the future. Third, the data collection of this study adopts the phased pairing method for the questionnaire survey. Future research can also be combined with paired samples, diversified data surveys, and other methods to reduce the impact of common method deviation.

## Data Availability Statement

The raw data supporting the conclusions of this article will be made available by the authors, without undue reservation.

## Author Contributions

WX: conceptualization, methodology, formal analysis, software, and writing – original draft preparation. X-LW: formal analysis, visualization, and supervision. Y-NC: data collection and writing – review and editing. All authors have read and agreed to the final version of the manuscript.

## Conflict of Interest

The authors declare that the research was conducted in the absence of any commercial or financial relationships that could be construed as a potential conflict of interest.

## Publisher’s Note

All claims expressed in this article are solely those of the authors and do not necessarily represent those of their affiliated organizations, or those of the publisher, the editors and the reviewers. Any product that may be evaluated in this article, or claim that may be made by its manufacturer, is not guaranteed or endorsed by the publisher.
